# The Alonissos Study: Cross-Sectional Study of the Healthcare Access and User Satisfaction in the Community of a Non-Profit-Line Greek Island

**DOI:** 10.3390/healthcare11131931

**Published:** 2023-07-04

**Authors:** Petros Kassas, Eudoxia Gogou, Charalampos Varsamas, Konstantinos Vogiatzidis, Aggeliki Psatha, Maria Pinaka, Dimitra Siachpazidou, Alexandra Sistou, Eleftherios D. Papazoglou, Despoina Kalousi, Konstantina Vatzia, Kyriaki Astara, Nikolaos Tsiouvakas, Sotirios G. Zarogiannis, Konstantinos Gourgoulianis

**Affiliations:** 1 Department of Respiratory Medicine, Faculty of Medicine, School of Health Sciences, University of Thessaly, Biopolis, 41110 Larissa, Greece; 2 Department of Physiology, Faculty of Medicine, School of Health Sciences, University of Thessaly, Biopolis, 41500 Larissa, Greece

**Keywords:** cross-sectional study, health service access, health service quality, insular public health, primary healthcare

## Abstract

Healthcare access and a high quality of the provided services to healthcare users are fundamental human rights according to the Alma Ata Declaration of 1978. Although 45 years have passed since then, health inequalities still exist, not only among countries but also within populations of the same country. For example, several small Greek islands have only a small Primary Healthcare Center in order to provide healthcare services to the insular population. In the current study, we investigated the level of self-reported overall, dental and mental health status and the level of satisfaction regarding the access to and the quality of the healthcare services provided by the Primary Healthcare center of Alonissos, along with registering the requirements for transportation to the mainland in order to receive such services. In this questionnaire-based cross-sectional study, 235 inhabitants of the remote Greek island of Alonissos that accounts for nearly 9% of the population participated (115 males and 120 females). The self-reported overall health status was reported to be moderate to very poor at a percentage of 31.49%, and the results were similar for dental and self-reported mental health status. Although nearly 60% of the participants reported very good/good quality of the healthcare provision, only 37.45% reported that the access to healthcare was very good/good, while around 94% had at least one visit to the mainland in order to receive proper healthcare services. Strategies for improving access to healthcare services need to be placed in remote Greek islands like Alonissos.

## 1. Introduction

Greek islands are numerous and mostly small in size, having, usually, only a Primary Healthcare Center (PHC) to meet the demands of inhabitants for healthcare provision. These PHCs ordinarily have personnel shortages, and the resources with which they operate are very limited, especially on distant islands that belong to the category of the non-profit line, which implies an infrequent connection to the mainland that is subsidized by the Greek state [1]. Globally, healthcare access is characterized by significant disparities at the individual and population levels, despite orchestrated efforts from several international and national organizations to reduce them [2]. Equity in healthcare access is best defined with respect to whether or not people who need medical treatment receive it [3]. The concept of a policy that emphasizes the notion of “health for all” is based on the principle that health constitutes a human right, requiring the eradication of health disparities based on scientific evidence. To assess the extent to which certain features of a population determine its access to healthcare, it is necessary to examine their characteristics and population-specific epidemiological traits [2]. In a representative Greek sample of 1000 participants with unmet needs in Primary Healthcare services, it was found that 10% reported a lack of satisfactory health provision [4]. Notably, it was found that individuals with primary and secondary education reported far more barriers in the accessibility and availability of health access compared to participants with tertiary education, while the same was the case for women [4]. Epidemiological studies are challenging the traditional belief that rural populations are healthier and live longer than urban dwellers [5]. More specifically, in a USA study featuring nearly half a million patients, it was shown that patients in rural clinics had significantly worse health status and primary healthcare utilization as compared to counterparts in urban clinics [5]. Furthermore, in a German cross-sectional study conducted in more than 2000 participants, urban participants were significantly more likely to report allergies as compared to farmers residing in rural areas [6]. Overall, recent evidence highlights that rural residents have increased all-cause mortality rates as compared to urban residents in the USA [7].

Chronic diseases, like diabetes and cancer, are clinical conditions associated with significant health disparities, while socioeconomic variables have been identified as modifiable risk factors [8]. In Greece, there is a scarcity of studies addressing the set of parameters framing the healthcare satisfaction levels in insular populations with respect to the prevalence of diseases like the ones mentioned above. This deters the establishment and application of population-specific health promotion strategies. Two recent studies by our research group highlighted the fact that insular smoking prevalence is substantially higher than the already-high national prevalence [9,10]. Moreover, in the recently published first report of the Alonissos Study, it was shown that there is a high prevalence of COPD and OSAS on Alonissos island, highlighting the need for primary healthcare awareness in this non-profit-line Greek island [1]. To this end, the aim of the present study was to address the level of healthcare access and user satisfaction in the population of the Alonissos Study.

## 2. Materials and Methods

### 2.1. Study Design and Enrollment

This cross-sectional study took place in the Healthcare Center of Alonissos Island, which is in Patitiri village and constitutes the only healthcare provision center on the island, from 5 July 5 through to 11 July 2018. The distance of the port of Alonissos from the port of Volos, which is the closest port connecting the island to the mainland, is 83 km (5.5 h by boat). The workforce of the Healthcare Center of Alonissos comprised 4 health professionals, more specifically, a rural medical doctor (who was a medical graduate that was assigned to this rural position straight after his graduation from the Medical School for a tenure of a year), a General Practitioner, a nurse and a technician for X-rays. Alonissos belongs to the Sporades complex of islands in the Aegean Sea and has a population of 2750 according to the 2011 Census [1]. The Community Centre of Alonissos Municipality informed the community on the screening dates and the scope of the research and arranged appointments of volunteers with the research team. The research team comprised 2 faculty members of the Faculty of Medicine of the University of Thessaly (S.G.Z., K.I.G), 1 General Practitioner (E.G), 2 respiratory medicine consultants (H.V., A.P.), 1 cardiologist consultant (K.V.), 3 nurses (D.S., M.P., A.S.), 1 graduate student (P.K.), and 5 undergraduate students of the Medical Faculty (E.D.P., D.K., K.V., K.A., N.T.). Written informed consent was obtained from each participant upon arrival at the Healthcare Center after they were fully informed about the study’s aim and procedure. A nurse collected the information regarding each participants’ medical history, followed by the provision of questionnaires. Each questionnaire had to be fully answered in order to be used for further analysis. Participants completed the questionnaires in the presence of a member of the research team that provided potential clarifications. Two hundred forty-five participants were enrolled in the study. Nine of them did not meet the inclusion criteria and were excluded. Participants were required to meet the following inclusion criteria: (a) age over 18 years, (b) permanent residence on the island (>6 months per year) and (c) possessing a driver’s license. One participant was excluded because he had not answered all the access to health service questions.

### 2.2. Assessment Tools—Questionnaires

Our study used two assessment tools: (a) a demographic data questionnaire for registering the population characteristics; (b) the health assessment questionnaire for self-reported health status, access to and quality of the provided health services [11]. This questionnaire was established in the Greek population in a set of female prisoners in 2016. The questionnaire was modified so it could refer to the community after excluding incarceration-related questions.

### 2.3. Statistical Analysis

Data were tabulated in an Excel spreadsheet, and statistical analysis was performed with GraphPad Prism v.9.3.1. Kolmogorov–Smirnov normality testing was performed, and two group comparisons were conducted with Student *t*-test or the Mann–Whitney U test for parametric and nonparametric data, respectively. When frequencies were compared, Chi-square test was performed. A *p* < 0.05 was considered statistically significant.

## 3. Results

### 3.1. Sample Characteristics

The final sample comprised 235 individuals (115 males and 120 females), corresponding to 8.54% of the total population of Alonissos island (which was 2750 inhabitants in the 2011 Greek Census). A detailed description of the demographic characteristics of the participants is provided in Table 1.

### 3.2. Self-Reported Health Status

Participants’ self-reported health status was assessed using a set of three questions. The first question referred to their overall health status, the second to their dental health and the third to their mental health. In all questionnaires, participants answered on a 5-point Likert scale. A detailed description of the self-reported community health status of our sample is provided in Table 2. 

Out of all participants, 161/235 (68.51%) reported very good or good overall health status, 42/235 (17.87%) moderate overall health status and 32/235 (13.62%) poor or very poor overall health status. Males reported statistically significantly better overall health status than females (*p* = 0.007). Stratifying the age of the population in the following groups, 18–34, 35–44, 45–54, 55–64, 65–74 and 75–92, it was found that there was a significantly worse overall health status in the higher age groups (*p* < 0.001). Stratifying for educational level, as shown in Table 1, it was found that a higher educational level improves overall health status (*p* < 0.001). 

Regarding dental health status, 141/235 (42.55%) reported it to be very good or good, 50/235 (21.28%) were moderate and 44/235 (18.72%) were poor or very poor. No statistically significant difference in dental health status was found among males and females (*p* = 0.172). Stratifying the age of the population in the following groups, 18–34, 35–44, 45–54, 55–64, 65–74 and 75–92, it was found that there was significantly worse dental health status in the higher age groups (*p* < 0.001). Stratifying for educational level, as shown in Table 1, it was found that a higher educational level improves dental health status (*p* < 0.001). 

Out of all participants, 152/235 (64.68%) reported very good or good mental health status, 49/235 (20.85%) moderate mental health status and 34/235 (14.47%) poor or very poor mental health status. Females reported statistically significantly worse mental health than males (*p* = 0.009). There was no statistically significant difference in the mental health status between males and females comparing age (*p* = 0.106) and educational level (*p* = 0.069).

### 3.3. Access to Healthcare Services and Quality

Participants’ opinions regarding the access to and the quality of healthcare services were assessed using a set of four questions. The first question referred to whether there was a need to travel outside Alonissos in order to receive appropriate healthcare. The second requested their opinion about the access to healthcare services provided in Alonissos; the third requested their opinion regarding the quality of healthcare services provided in Alonissos; the fourth requested their opinion regarding whether it was possible to travel away from Alonissos in case of an emergency medical situation. In all four questions, participants answered on a 5-point Likert scale. A detailed description of the self-reported community health status in our sample is in Table 3.

It can be seen from the data in Table 3 that the opinions of females about access to healthcare were significantly worse than the opinion of male counterparts (*p* < 0.001). On the other hand, no significant difference was found regarding age (*p* = 0.750) and educational level (*p* = 0.394). Females had a significantly worse opinion about the quality of healthcare services than males (*p* = 0.030), whereas no significant difference was found relative to age (*p* = 0.112) and educational level (*p* = 0.107). Moreover, no significant difference was found regarding sex (*p* = 0.777), age (*p* = 0.304) and educational level (*p* = 0.241).

The participants reported that they were obliged to travel to the mainland in order to seek medical and health services from various specialists due to the lack of such an option in Alonissos, as demonstrated in Table 4. 

An important finding in our study was that there was a significant correlation of the self-reported general, dental and mental health with the access to health services, as shown in Table 5. In this analysis, it was evident that the better perception of the participants’ health correlated with a better perception of access to the health services provided in Alonissos.

Likewise, there was a significant correlation of the self-reported general, dental and mental health with the quality of health services, as shown in Table 6. Better perception of the participants’ health correlated with a better perception of the quality of the health services provided on the island.

## 4. Discussion

This study investigated the general population of Alonissos, an island in Northern Sporades, that belongs to the underprivileged non-profit-line islands of Greece. We were able to screen nearly one-tenth of the island population (8.54%) that renders credibility to our results regarding self-reported healthcare access and quality. 

Approximately 13.62% of the participants reported poor or very poor health status in our study, and this result corroborates previous findings in Europe [11,12]. More specifically, according to a study that examined the self-reported health status of European residents from 28 European countries, including Greece, 14% reported poor or very poor overall health status in 2007, and the corresponding percentage slightly declined in 2012 to 13% [12]. Another study that was conducted on a population of female Greek prisoners had similar results since 13.9% of the participants reported poor or very poor overall health status [11]. Our findings also suggest that people with a higher educational level had a better self-reported overall health status. Indeed, a Greek health survey in 2010 reported that the socioeconomic status in the Thessaly prefecture of Greece was independently associated with the health-related quality of life, and, more specifically, individuals with primary or secondary education reported poorer general health status compared to those with university-level education [13]. The findings of another study focusing on 11 European Union countries, including Greece, were similar. There was a positive impact of secondary and tertiary education on health status, and the mechanistic explanation provided was that education improves health literacy, that is, the degree to which individuals understand health information [14]. Indeed, a study in the Balkan area found that inhabitants of rural areas were more likely to show inadequate and marginal health literacy compared to those of urban areas [15].

Another important finding of our study was that the health services on Alonissos were not adequate to fulfill the health needs of the community, since more than two-thirds of the participants had a negative opinion about the accessibility to healthcare services. A key issue policymakers and stakeholders must address in the future in order to ensure reasonable access to PHCs in rural and remote areas is the issue of availability and geography [16]. Indeed, in our study, the challenging availability and geography were evident since 9 out of 10 participants had to travel away from Alonnisos at least once in their life to have access to the health services that they needed. These findings suggest that PHC services were not sufficiently available for Alonnisos healthcare users. Additionally, although PHC services are free of charge in Greece, one should take into account the significant costs related to traveling to the mainland for healthcare services along with the costs related to absence from work. Therefore, another challenge that stakeholders and policymakers have to tackle with respect to insular rural areas in Greece would be to attract and retain healthcare personnel [17,18]. There are specific interventions that policymakers can employ in order to achieve this by strengthening rural training pathways [17]. 

Even though nearly half of the participants reported being able to travel to a mainland hospital when required, islanders face delays in receiving care due to travel time, particularly in urgent situations. In addition, almost half of the participants were positive regarding the quality of healthcare in Alonissos, although one’s perception of the quality of healthcare is influenced by his or her attitudes toward health and illness and also depending on the residence status (urban or rural) [19]. Moreover, we demonstrated that there is a significant correlation of the self-reported health perception with the perception of access and quality to health services. In rural areas, health awareness is of particular importance since people have less health information available through primary care providers, specialist doctors, blogs, magazines and internet search engines. On the other hand, rural dwellers have greater difficulty accessing mass media and scientific literature, and this has a negative impact on health literacy as well as the attitude of the population toward their health [20,21,22].

## 5. Conclusions

In conclusion, the majority of the insular population of Alonissos reported above-average, overall, dental and mental health status. A percentage of 13.6% that is in accordance with the published literature reported poor/very poor health status. Although nearly 60% of the participants reported very good/good satisfaction with the quality of the healthcare provided on the island, only 37.45% reported that the access to healthcare was very good/good and, furthermore, around 94% of the participants had at least traveled to the mainland at least once in order to receive proper healthcare services. Considering that, in the rural population under study, the only available healthcare service is the PHC of Alonissos, a detailed epidemiological investigation of the prevalence of non-communicable diseases is necessary for targeted health promotion and disease prevention strategies.

## Figures and Tables

**Table 1:** Sociodemographic characteristics of the participants.

	Males	Females	*p* Value
Gender (*n*)	115 (48.94%)	120 (51.06%)	
Age (years)	56 (42, 73)	54.50 (38.25, 67.75)	0.210
BMI (kg/cm^2^)	27.70 (26.68, 30.52)	25.50 (22.59, 29.26)	0.003
Education (*n*)			
No education	3	5	0.420
Primary	35	39	
Secondary	24	14	
Post-Secondary	5	12	
Tertiary	26	23	

Age and BMI are presented as the median value (25th and 75th quartiles).

**Table 2:** Self-reported health status.

	Very Good /Good	Moderate	Poor /Very Poor
Overall Health (*n*)	161 (68.51%)	42 (17.87%)	32 (13.62%)
Dental Health (*n*)	141 (60.00%)	50 (21.28%)	44 (18.72%)
Mental Health (*n*)	152 (64.68%)	49 (20.85%)	34 (14.47%)

**Table 3:** Healthcare access and quality.

		Community (%)	Males (%)	Females (%)	*p* Value
Was there ever a need to travel outside Alonissos to receive a health care service?	Yes	93.19	92.17	93.33	0.804
	No	6.81	7.83	6.67	
How would you describe the access to Health Care in Alonnisos?	Very good/Good	37.45	51.30	24.17	<0.001
	Moderate	20.43	16.52	24.17	
	Poor/Very poor	42.13	32.17	51.67	
How would you describe the quality of Health Care in Alonnisos?	Very good/Good	59.15	67.83	50.83	0.13
	Moderate	25.53	20.00	30.83	
	Poor/Very poor	15.32	12.17	18.33	
Was transport to a hospital outside Alonissos possible when necessary?	Yes	49.36	50.43	47.90	0.78
	No	17.45	15.65	19.33	
	Not Needed	33.19	33.92	32.77	

**Table 4:** Visits of participants to healthcare providers in the mainland demonstrated by specialty.

Specialty	Community (%)	Males (%)	Females (%)
Dentist	60.85	60.00	62.5
Internal Medicine	45.96	53.04	39.17
Gynecologist	37.45	0.00	72.73
Cardiologist	37.02	37.39	36.67
General Surgeon	34.47	37.07	31.40
Microbiologist	25.96	20.00	31.67
Dermatologist	22.98	18.26	27.50
Gastroenterologist	19.15	18.26	20.00
Ophthalmology	16.17	14.78	17.50
Otorhinolaryngologist	14.47	14.78	14.17
Physiotherapist	13.62	14.78	12.5
Urologist	8.09	16.52	0.00
Psychiatrist	7.66	5.22	10.00
Pulmonologist	5.96	3.48	8.33
Neurologist	3.40	2.61	4.17
Endocrinologist	1.70	0.87	2.5
Rheumatologist	1.28	0.00	2.50
Angiologist	0.85	0.87	0.83
Hematologist	0.85	0.00	1.67
Neurosurgeon	0.85	0.87	0.83
Oncologist	0.85	0.87	0.83
Nephrologist	0.43	0.00	0.83

**Table 5:** Correlation of self-reported health and access to health services.

		Very Good/Good	Moderate	Poor/Very Poor	*p*
Self-reported general health	Very Good/Good	76.14%	12.50%	11.36%	0.05
	Moderate	74.47%	19.15%	6.38%	
	Poor/Very poor	58.59%	22.22%	19.19%	
Self-reported dental health	Very Good/Good	65.91%	21.59%	12.50%	0.01
	Moderate	57.45%	27.66%	14.89%	
	Poor/Very poor	44.30%	22.79%	32.91%	
Self-reported mental health	Very Good/Good	78.41%	14.77%	6.82%	<0.001
	Moderate	55.32%	36.17%	8.51%	
	Poor/Very poor	56.57%	19.19%	24.24%	

**Table 6:** Correlation of self-reported health and quality of health services.

		Very Good/Good	Moderate	Poor/Very Poor	*p*
Self-reported general health	Very Good/Good	76.14%	12.50%	11.36%	0.05
	Moderate	74.47%	19.15%	6.38%	
	Poor/Very poor	58.59%	22.22%	19.19%	
Self-reported dental health	Very Good/Good	64.49%	23.19%	12.32%	<0.001
	Moderate	59.32%	22.03%	18.64%	
	Poor/Very poor	41.67%	13.89%	44.44%	
Self-reported mental health	Very Good/Good	76.81%	18.84%	4.35%	<0.001
	Moderate	52.54%	30.51%	16.95%	
	Poor/Very poor	36.11%	13.89%	50.00%	

## Data Availability

Data are available from the corresponding author upon reasonable request.
